# The distinct clinical profile of chronically critically ill patients: a cohort study

**DOI:** 10.1186/cc4941

**Published:** 2006-06-19

**Authors:** Elisa Estenssoro, Rosa Reina, Héctor S Canales, María Gabriela Saenz, Francisco E Gonzalez, María M Aprea, Enrique Laffaire, Victor Gola, Arnaldo Dubin

**Affiliations:** 1 Servicio de Terapia Intensiva, Hospital Interzonal General San Martín, La Plata, Buenos Aires, Argentina; 2 Critical Care Unit, Sanatorio Otamendi y Miroli, Buenos Aires, Argentina

## Abstract

**Introduction:**

Our goal was to describe the epidemiology, clinical profiles, outcomes, and factors that might predict progression of critically ill patients to chronically critically ill (CCI) patients, a still poorly characterized subgroup.

**Methods:**

We prospectively studied all patients admitted to a university-affiliated hospital intensive care unit (ICU) between 1 July 2002 and 30 June 2005. On admission, we recorded epidemiological data, the presence of organ failure (multiorgan dysfunction syndrome (MODS)), underlying diseases (McCabe score), acute respiratory distress syndrome (ARDS) and shock. Daily, we recorded MODS, ARDS, shock, mechanical ventilation use, lengths of ICU and hospital stay (LOS), and outcome. CCI patients were defined as those having a tracheotomy placed for continued ventilation. Clinical complications and time to tracheal decannulation were registered. Predictors of progression to CCI were identified by logistic regression.

**Results:**

Ninety-five patients (12%) fulfilled the CCI definition and, compared with the remaining 690 patients, these CCI patients were sicker (APACHE II, 21 ± 7 versus 18 ± 9 for non-CCI patients, *p *= 0.005); had more organ dysfunctions (SOFA 7 ± 3 versus 6 ± 4, *p *< 0.003); received more interventions (TISS 32 ± 10 versus 26 ± 8, *p *< 0.0001); and had less underlying diseases and had undergone emergency surgery more frequently (43 versus 24%, *p *= 0.001). ARDS and shock were present in 84% and 83% of CCI patients, respectively, versus 44% and 48% in the other patients (*p *< 0.0001 for both). CCI patients had higher expected mortality (38% versus 32%, *p *= 0.003), but observed mortality was similar (32% versus 35%, p = 0.59). Independent predictors of progression to CCI were ARDS on admission, APACHE II and McCabe scores (odds ratio (OR) 2.26, *p *< 0.001; OR 1.03, *p *< 0.01; and OR 0.34, *p *< 0.0001, respectively). Lengths of mechanical ventilation, ICU and hospital stay were 33 (24 to 50), 39 (29 to 55) and 55 (37 to 84) days, respectively. Tracheal decannulation was achieved at 40 ± 19 days.

**Conclusion:**

CCI patients were a severely ill population, in which ARDS, shock, and MODS were frequent on admission, and who suffered recurrent complications during their stay. However, their prognosis was equivalent to that of the other ICU patients. ARDS, APACHE II and McCabe scores were independent predictors of evolution to chronicity.

## Introduction

A growing population of patients (5% to 10%) survive acute critical illness only to become chronically critically ill (CCI), with profound weakness and ongoing respiratory failure [1]. The most perceptible feature of this group is their prolonged dependence on mechanical ventilation (up to 7% to 15% of intensive care unit (ICU) patients) [2]; however, chronic critical illness might be considered as a far more complex syndrome, characterized by physiological, metabolic, immunological, neuroendocrine and neuromuscular disturbances [1]. Repeated episodes of sepsis are the hallmark of the CCI, and possibly contribute to lengthened ICU stay.

The CCI are expected to increase, since more patients with complex diseases survive due to advances in resuscitation techniques, mechanical ventilation protocols, metabolic control and treatment of sepsis [3-6]. The use of invasive procedures and devices, and the presence of malnutrition and immunosuppression are well-known risk factors for infection in the ICU and are especially frequent in the CCI [7]. Despite prolonged and resource-intensive care, these patients suffer early mortality or slow recovery, with excessive functional dependency. In addition, the cost of their care is seemingly high, which has recently focused attention on their characteristics and outcomes [8]. Since data about this group are scarce, there is a plea to continuing research on them [9].

There is some controversy about who should be considered a CCI patient. Some overlap with the definition of prolonged mechanical ventilation (mechanical ventilation ≥21 days) seems unavoidable [2]. Notwithstanding this, other investigators have proposed the placement of a tracheotomy as a marker for chronic critical illness, given that it foresees the need for long-lasting ventilatory support [8]. In addition, the analysis of the events occurring during the whole length of ICU stay – and not just the days spent on mechanical ventilation – might yield a more comprehensive approach to the CCI, since there are other non-ventilatory, severe conditions that might also require prolonged ICU care (airway problems, parenteral nutrition, complicated wound care or continuous hemofiltration).

Our goal was to describe the epidemiological features, clinical complications, infectious profiles, and the outcomes of this recently defined, poorly characterized subgroup of ICU patients. In addition, we sought for independent predictors of evolution to chronic critical illness.

## Materials and methods

### Study design

This is a prospective cohort study carried out in a medical-surgical ICU located in a university-affiliated hospital of 474 beds in La Plata, Buenos Aires, Argentina. This study was approved by the Institutional Review Board. Informed consent was waived since no interventions on patients would be performed.

### Patients

Patients admitted to the ICU between 1 July 2002 and 30 June 2005 that ultimately required a tracheotomy placed for continued ventilation were considered CCI [9]. Patients with tracheotomies performed for other conditions, such as maxilo-facial trauma or laryngeal surgery, were excluded. Tracheotomies are performed in our ICU based on an expectation of a prolonged mechanical ventilation course. CCI patients usually remain in the hospital until death or weaning, because health systems in Argentina do not provide readily accessible long-term acute care facilities for their transfer.

For the whole population, we prospectively recorded: age, gender, main diagnosis, primary source of admission (emergency room, hospital ward, other hospital), severity of illness (Acute Physiologic and Chronic Health Evaluation (APACHE) II score) [10] and predicted mortality on admission; intensity of procedures and organ failures (therapeutic intervention scoring system (TISS)_24 hs _and sequential organ failure assessment (SOFA)_24 hs _scores) during the first day of admission [11,12]; type of admission (medical, emergency or elective surgery); causes of initiation of mechanical ventilation (respiratory, hemodynamic, neurological and postoperative) [13]; and pre-existent illnesses (McCabe score) as non-fatal (score of 1), ultimately fatal (score of 2) or rapidly fatal disease (score of 3) [14].

Every day, we screened all patients for the presence of: acute respiratory distress syndrome (ARDS; defined as an acute onset respiratory failure, with an arterial oxygen partial pressure/inspired oxygen concentration relationship (PaO_2_/FiO_2_) ≤200 and bilateral infiltrates on chest X-ray, in the absence of a wedge pressure >18 mmHg or clinical heart failure) [15]; shock (defined as systolic blood pressure <90 mmHg or a reduction of >40 mmHg from baseline despite adequate fluid resuscitation, along with the presence of perfusion abnormalities that might include oliguria, lactic acidosis, or acute altered mental status) [16]; septic shock [16]; and causes for initiation of mechanical ventilation [13], if newly started.

When a patient became tracheotomised, we prospectively recorded the following complications daily and during the entire length of ICU stay: organ dysfunctions; new episodes of shock; respiratory events; neuromuscular events; neuropsychological events; gastrointestinal and metabolic events; use of hemodyalisis; and infectious complications. In addition, we made a retrospective search for these complications in the period that extended from the admission day to the day of tracheotomy by reviewing clinical records and charts. Data presented correspond to the whole length of stay.

#### Organ dysfunctions

Organ dysfunctions (MODS) were defined as a SOFA score >2 points in at least two organ systems (cardiovascular, respiratory, neurological, hematological, renal hepatic).

#### Respiratory events

Respiratory events included: the development of ARDS; episodes of atelectasis, defined as pulmonary infiltrates that resolve within 48 hours of bronchoscopy, recruitment maneuvers or chest physiotherapy, and no other etiology to account for it [17]; a number of failed weaning attempts, defined as the inability to sustain a spontaneous breathing trial of two hours [18]; unplanned extubations [19]; extubation failures, defined as reintubation within 48 hours; days to tracheotomy; decannulation failures, defined as need for re-cannulation with a tracheostomy tube after a decannulation attempt, due to airway obstruction or to inability to swallow without aspiration of liquid/food contents into the airway, evaluated either clinically or by fiberoptic endoscopy [20]; days to successful decannulation; and duration of mechanical ventilation (LOS_MV_), defined as effective days on mechanical ventilation (days spent on T tube during finally unsuccessful weaning attempts were not considered as mechanical ventilation days).

#### Neuromuscular events

Neuromuscular events comprised the development of critical illness polyneuropathy/myopathy. When sedative-analgesics were withdrawn, patients were assessed for the presence of muscular weakness. An electromyogram was then performed to confirm the diagnosis. Infusion of neuromuscular blocking agents lasting longer than 24 hours was also recorded.

#### Neuropsychological events

Neuropsychological events constituted the presence of intracranial hypertension, or psychomotor agitation, defined as the presence of restlessness, anxiety, agitation or combativeness (Ramsay sedation scale = 1) [21].

#### Gastrointestinal and metabolic events

Gastrointestinal and metabolic events recorded included clinically evident upper gastrointestinal hemorrhage (altering hemodynamics or requiring transfusion), diarrhea, ileus, days of enteral nutrition, interruptions and its causes: patient-related (shock or intolerance) or nasogastric-tube related (obstruction, malposition, accidental withdrawal) and days of parenteral nutrition.

#### Infectious complications

Infectious complications were considered when fever or hypothermia evolved, in which case cultures were taken and sites of infection diagnosed as ventilator-associated pneumonia (VAP), catheter-related infections, primary bacteremias, and urinary tract infections. VAP was the presence of a new or persistent radiographic infiltrate occurring > 48 hours after mechanical ventilation onset [22], plus purulent tracheal secretions, plus either a positive quantitative secretion culture yielding ≥10^4 ^cfu/ml in bronchoalveolar lavage [22], or ≥10^3 ^cfu/ml in mini-bronchoalveolar lavage [23] or ≥10^6 ^cfu/ml in tracheal aspirate [24]. Catheter-related infections constituted ≥15 cfu in a semi-quantitative, or ≥10^3 ^cfu in a quantitative, culture from a catheter tip, and/or exit-site infection plus isolation of the same microorganism from blood drawn from a peripheral vein and no other apparent source of infection [25]. Primary bacteremias were at least one positive blood culture without another site simultaneously infected with the same microorganism [26]. Urinary tract infections were pyuria (≥10^5 ^leucocytes/mm^3^) plus urine culture ≥10^5 ^cfu/ml [26].

Isolated microorganisms, absolute, relative number of infectious episodes, frequency of polymicrobial infections, and days to first episode and crude mortality for each type of infection were registered. Incidence densities (episodes per 1,000 days ICU stay or device use for mechanical ventilation, catheters, urinary catheters) were calculated and compared to those of non-CCI patients.

After death or discharge, ICU and hospital stay (LOS_ICU _and LOS_Hosp_) were recorded.

### Outcome variables

The main outcome variable for the entire population was the evolution to chronic critical illness. For CCI patients, it was hospital mortality.

Do-not-resuscitate orders were not explicitly recorded, since this is an infrequent practice in countries of Latin origin [27].

### Statistical analysis

Results are expressed as percentages, mean ± standard deviation for continuous parametric variables, and median and interquartile range (25% to 75% IQ) for continuous, non-parametric variables. Comparisons between groups were performed by the use of Chi-square, *t*, and Mann-Whitney tests, as appropriate. A *p *value of less than 0.05 was considered significant. Variables that differed between CCI and non-CCI patients on admission (*p *< 0.10) were tested for interaction with multiple logistic regression analysis, looking for independent predictors of evolution to chronicity. Odds ratios (ORs) and 95% confidence intervals were calculated. A *p *value < 0.05 was considered significant. Discrimination of the logistic regression model was assessed using the area under the receiver operator characteristic curve.

All statistical analyses were performed with statistical software (Stata 8.0; Stata Corporation, College Station, TX, USA).

## Results

### Comparison of CCI versus the rest of the patients

During the study period, 785 patients were admitted to the ICU; 95 patients (12%) were considered CCI. Only 21 (3%) of the remaining 690 patients had a tracheotomy placed for other reasons (13 for head and neck trauma/surgery, 8 for otorhinolaryngological diseases). Characteristics of both groups of patients are shown in Table 1. Of note, CCI patients were significantly more acutely ill on admission, according to prognostic, organ dysfunction and intervention scores, and to the prior requirement for emergency surgery. Recurring episodes of ARDS and shock were the rule in CCI patients during their ICU stay. Interestingly, underlying diseases were significantly more common in the non-CCI patients. As expected, CCI patients had a protracted course of disease, demonstrated by a longer duration of mechanical ventilation and ICU and hospital LOS. Despite this, the observed hospital mortality of 32% for CCI patients was lower than expected, and similar to non-CCI patients.

Of the variables that differed between CCI and non-CCI patients, ARDS on admission (OR 2.26, *p *< 0.001), APACHE II (OR 1.03 per point, *p *< 0.01) and McCabe score (OR 0.34, *p *< 0.0001) independently predicted evolution to chronic critical illness. Discrimination of the model was fair (the area under the receiver operator curve was 0.74).

### Characterization of CCI patients and comparison between survivors and non-survivors

CCI patients showed high rates of hemodynamic, respiratory, gastrointestinal, nutrition-related and neuropsychological events (Tables 2 and 3). Perioperative shock on admission (32% of shock cases), and septic shock developing during ICU stay (95% of cases) were the most common causes of hemodynamic dysfunction.

On admission, CCI survivors and non-survivors had similar severity of illness, organ failures and high rates of ARDS and shock (Table 2). At discharge/death, they also had similar lengths of mechanical ventilation and stay at the ICU. The variables that differed between survivors and non-survivors were age and having been transferred from another hospital, and the number of shock episodes showed significant differences between them (Table 2). In addition, the non-survivors showed an increased frequency of MODS (94% versus 68% in survivors; *p *< 0.01), upper gastrointestinal hemorrhage (29% versus11%, *p *= 0.03), ileus (25% versus 48%, *p *< 0.02), and longer duration of parenteral nutrition (58 ± 34 versus 12 ± 7, *p *< 0.02). Other variables, such as weaning attempts and successful and failed decannulations, were, expectably, more frequent in surviving patients.

A detailed description of events in the whole CCI population is given in Table 3.

Infectious episodes were recurrent and showed an important crude mortality (Table 4). Primary bacteremia (of unidentified source) was the most frequent single infection type, with a rate of 1.3 episodes per patient rate. Prevalent microorganisms found were: Gram-negative bacilli in 63% of primary bacteremias (45% Enterobacteriaceae); *Acinetobacter *and *Pseudomonas *in 28 and 22% of VAPs; *Candida *in 70% of urinary-tract infections; and Gram-negative bacilli in 45% of catheter-related infections.

## Discussion

The main contributions of this study refer to the characterization of a growing subgroup of ICU patients, the CCI, in terms of frequency, prognostic factors and outcome.

The CCI patients represented 12% of admissions, and were similar to the remaining patients in age and gender. However, they were more acutely ill from the start – previous comorbid states were uncommon – and had higher predicted mortality than non-CCI patients (38% versus 32%). Surprisingly, evolution to chronically critical illness did not lead to a worse prognosis: mortality rate was 32% and 35% for CCI and non-CCI patients, respectively. Most studies on CCI have been done in post-ICU settings, so comparison is difficult [28-30]; however, reported mortality rates lie between 29% and 51%. In two studies enrolling ICU patients undergoing prolonged mechanical ventilation, mortality rates have been as high as 71% and 43% [31,32], but these figures might have changed due to recent advances produced in the critical care arena [3-6]. More recently, ICU mortalities of 25% [33] and 44% [34] have been reported for patients undergoing mechanical ventilation for more than 14 days, and in an international study of mechanical ventilation, hospital mortality of tracheostomised patients equaled that of non-tracheostomised patients (39% versus 40%) [35].

Interestingly, reported one to three year survival rates are good [28-30], with an acceptable health-related quality of life [31]. These are encouraging results in the face of the elevated numbers of complications CCI patients do develop.

There are no available models to predict progression of ICU patients to chronically critical illness [1]. Indeed, we identified admission variables as ARDS, APACHE II and absence of significant comorbidities as independent risk factors for evolution to CCI.

Severity scores have been repeatedly identified as predictors of prolonged mechanical ventilation [36,37]. This condition and chronically critical illness might have common characteristics, and so share some determinants. However, the presence of ARDS and the absence of underlying disease as predictors of chronicity in the ICU are novel findings.

This last result requires further discussion. CCI patients were more acutely ill, but had less underlying diseases than the non-CCI. Mortality was equal for both. Perhaps, with less comorbidity, the CCI patients had a greater physiological reserve that allowed them not to die. Instead, they developed a chronic course of disease with plenty of complications.

It is difficult to ascertain whether complication rates were a consequence of the prolonged exposure to the ICU milieu, or of the complex pathophysiological features of chronic critical illness [1,8,9]. In any case, event rates might need considerable adjustment before comparing them to those of other ICU populations [38].

### Respiratory events

As expected [39], many unsuccessful weaning attempts (11 ± 12 per patient) occurred in this chronic, debilitated and multi-infected population. Atelectasis (in 35% of patients), malnutrition and critical illness polyneuropathy/myopathy could certainly account for that.

Tracheotomy was performed at 16 ± 6 days, and was preceded by 26% of extubation failures, which have been linked to increased mortality when caused by airway-unrelated causes [40]. Tracheal decannulation was a late achievement, performed 40 ± 19 days after tracheotomy (one week after weaning). Previous failures were observed in 23% of patients. This reflects other neuromuscular disturbances that can prolong ICU stay – ineffective cough and swallowing dysfunction.

### Neuromuscular and neuropsychological events

Critical illness polyneuropathy/myopathy, another manifestation of the systemic response inflammatory syndrome [41], occurred in 16% of patients. Axonal polyneuropathy was the most frequent electromyogram diagnosis (13%), performed approximately at day 25. More subtle forms might have gone unrecognized. Psychomotor agitation occurred in only 16% of patients, but this figure may not include minor alterations. The relatively young age of our cohort might explain the relatively low incidence of this complication.

### Gastrointestinal and metabolic events

Stress-related mucosal damage and gastrointestinal hemorrhage occurs in up to 25% of critically ill patients [42]. Notwithstanding the use of prophylactic therapy, clinically significant bleeding occurred in 17% of patients, and was significantly more frequent in non-survivors. It has been associated with increases in mortality and in ICU stay of as much as 11 days [43].

Interruptions of enteral nutrition were common, and mostly related to shock. Ileus and diarrhea were frequent; however, intolerance to enteral nutrition might act as a marker of severity of illness [44].

### Infectious events

Not unexpectedly, CCI patients have very high infection rates, related to the exposure of multiple entry sites to a highly colonized environment during long periods, intense antimicrobial use, prolonged mechanical ventilation, parenteral nutrition, cognitive impairment and compromised immune function.

Other particular characteristics of CCI patients explain the high rate of some infections. Key risk factors for VAP are present in this cohort: the use of mechanical ventilation during long periods, which leads to an increasing incidence of VAP until the 30th day, at which it plateaus [44]; and the great incidence of ARDS (present in 83% of patients), which has been found to precede VAP in 34% to 70% of patients [45].

Primary Gram-negative bacilli bacteremia was the most frequent infection in CCI patients, and was more than double the incidence in the rest of the patients. Catheter related infections displayed similar behavior. Recurrent episodes of infection and septic shock were frequent. Each type of infection displayed a distinctive microorganism pattern, and polymicrobial isolations were common. These were late events, appearing at a median of 17 (11 to 39) days, and might help to identify appropriate empiric antimicrobial therapy, which has been associated with better outcomes [46].

However, overall infection rates might be confounded by the average LOS in the ICU [38], and also might just be an indicator of greater severity of illness. Larger hospitals have higher rates of device utilization, particularly ventilator use, which may lead to increased overall infection rates in their ICUs. However, the use of cumulative incidences (normalizations to events per 1,000 days of device use) might aid in comparisons with other patient groups. Discharge policies, for example, some ICUs can readily discharge CCI patients to step-down facilities, may lower infection rates. Both variables (large bed number, but lack of long-term acute care units) might have affected the results in our study.

A strength of the current study is the prospective gathering of data – only a small number of events were recorded retrospectively but, even so, patients were in the ICU when tracheostomy was performed, so the chance of having missed retrospective data was small. Clinical complications were assessed with standard definitions. In addition, the number of patients is half that reported in a population-based cohort of patients with a long ICU stay recently published [33]. The percentage of tracheostomised patients is identical to that of the European group of the International Mechanical Ventilation Study Group [35]. The big number of events – reported for the first time with such detail – gives a global picture of the problem of chronic patients in the ICU. This study also identifies new factors associated with progression to chronicity.

The limitations of the present study are related to its observational design. Selection bias is possible, since physicians are prone to tracheostomise patients that are expected to survive; the 'protection' McCabe score seems to suggest (OR 0.34 in the logistic regression model) points in this direction. Another limitation refers to the generalizability of these results, since it was carried out in a single ICU. Finally, practice patterns might have changed during the three-year period this study took. However, the main advances in critical care had already been published [3-6] when the study started, so procedures and therapeutics might not have changed further. Discharge policies were also maintained, since no new long-term acute care units were opened in the area served by the hospital, nor was the possibility of home care of the CCI accepted by most health payers.

## Conclusion

CCI patients are a distinct, acutely and severely ill population in which ARDS, shock and organ failure are extremely frequent from admission onwards, and who suffer recurrent respiratory, infectious, gastrointestinal and neuropsychological complications during the course of their illness. The high frequency of atelectasis, extubation failures, unsuccessful weaning attempts, malnutrition and infections might explain their prolonged ICU stay. Even though hospital mortality is high, it is not different from that of other admitted patients, and even lower than expected. ARDS, APACHE II and the absence of significant underlying diseases on admission independently predicted the evolution to chronic critical illness.

## Key messages

• 	CCI patients suffer many pathophysiological disturbances and clinical complications, but overall prognosis is not different from other ICU patients.

• 	ARDS on admission, APACHE II and absence of significant comorbidities predict progression to chronicity.

## Abbreviations

APACHE = Acute Physiologic and Chronic Health Evaluation; ARDS = acute respiratory distress syndrome; CCI = chronically critically ill; ICU = intensive care unit; LOS = length of stay; MODS = multiorgan dysfunction syndrome; SOFA = sequential organ failure assessment; TISS = therapeutic intervention scoring system; VAP = ventilator-associated pneumonia.

## Competing interests

The authors declare that they have no competing interests.

## Authors' contributions

EE was responsible for the study concept and design, analysis and interpretation of data, revised the manuscript critically for important intellectual content and gave final approval of the version to be published. RR and AD participated in the conception and design of the study. EL participated in the design of the study and performed the statistical analysis. FEG, HSC, MGS, MMA and VG performed acquisition of data and contributed to drafting of the manuscript. All authors read and approved the final manuscript.

## Figures and Tables

**Table 1:** Epidemiological, clinical and outcome variables in CCI patients compared to the rest of ICU admissions

	CCI	Non-CCI	*P *value
*n*	95	690	
Age (years)	44 ± 16	41 ± 18	0.21
Gender (male)	50 (53)	356 (52)	0.76
APACHE II	21 ± 7	18 ± 9	0.005
APACHE II mortality risk	38%	32%	0.003
Mortality	30 (32)	240 (35)	0.59
SOFA 24 hours	7 ± 3	6 ± 4	0.003
McCabe score	1.2 ± 0.5	1.6 ± 0.8	<0.0001
TISS score	32 ± 10	26 ± 8	<0.0001
ARDS			
At any point	80 (84)	304 (44)	<0.0001
On admission	52 (55)	229 (33)	0.0001
In evolution^b^	72 (76)	157 (23)	<0.0001
Shock			
At any point	79 (83)	331 (48)	<0.0001
On admission	58 (61)	286 (42)	<0.001
In evolution^b^	68 (72)	180 (26)	<0.0001
Cranial trauma	19 (20)	88 (13)	0.05
Admission type			
Medical	47 (50)	360 (52)	0.70
Emergency surgery	40 (43)	164 (24)	0.001
Elective surgery	8 (9)	158 (23)	0.001
Mechanical ventilation	94 (100)	355 (51)	<0.0001
LO_MV_(days)^a^	33 (24–50)	5 (2–9)	<0.0001
LOS_ICU _(days)^a^	39 (29–55)	4 (2–8)	<0.0001
LOS_Hospital _(days)^a^	55 (37–84)	17 (8–53)	<0.0001

Data are shown as *n *(%), or mean ± standard deviation, unless specified. ^a^Median and 0.25 to 0.75 interquartile range. APACHE, Acute Physiologic and Chronic Health Evaluation; ARDS, acute respiratory distress syndrome; CCI, chronically critically ill; Evolution^b^, developed during ICU stay; ICU, intensive care unit; LO_MV_, length of mechanical ventilation; LOS_Hospital_, length of stay at the hospital; LOS_ICU_, length of stay at the ICU; SOFA, sequential organ failure assessment; TISS, therapeutic intervention scoring system.

**Table 2:** Admission and clinical characteristics of CCI survivors and non-survivors

	Survivors	Non-survivors	*P *value
*n*	64	31	
Age (years)	40 ± 16	51 ± 12	0.0008
Gender (male)	35 (55)	15 (48)	0.56
APACHE II	20 ± 7	21 ± 6	0.51
APACHE II mortality risk (%)	37	42	0.33
SOFA 24 hours	7 ± 3	8 ± 3	0.28
McCabe score	1.2 ± 0.5	1.3 ± 0.6	0.38
TISS	32 ± 9	32 ± 19	0.81
Pre-admission			
Emergency room	38 (59)	16 (51)	0.47
Hospital ward	12 (19)	5 (16)	0.75
Transfer from other hospital	26 (41)	6 (19)	0.04
LOS previous hospitals (days)	6 ± 8	6 ± 8	0.97
Cause of mechanical ventilation			
Respiratory	19 (30)	9 (29)	0.95
Hemodynamic	23 (36)	9 (29)	0.50
Neurological	13 (20)	6 (19)	0.91
Postoperative	9 (14)	7 (23)	0.24
MODS	42 (68)	29(94)	0.01
ARDS			
On admission	35 (55)	17 (55)	0.98
In evolution^b^	48 (75)	24 (77)	0.79
Shock			
On admission	39 (61)	19 (61)	0.97
In evolution^b^	45 (70)	23 (77)	0.46
Number of shock episodes	59	55	0.05
LOS_ICU _(days)^a^	38 (29–53)	44 (30–58)	0.34
LOS_Hospital _(days)^a^	65 (42–83)	50 (32–62)	0.05

Data are shown as n (%), or mean ± standard deviation, unless specified. ^a^Median and 0.25 to 0.75 interquartile range. APACHE, Acute Physiologic and Chronic Health Evaluation; ARDS, acute respiratory distress syndrome; CCI, chronically critically ill; Evolution^b^, developed during ICU stay; ICU, intensive care unit; LO_MV_, length of mechanical ventilation; LOS_Hospital_, length of stay at the hospital; LOS_ICU_, length of stay at the ICU; SOFA, sequential organ failure assessment; TISS, therapeutic intervention scoring system.

**Table 3:** Events, complications and outcomes in CCI patients

*n*	95
MODS	71 (76)
Hemodyalisis	8 (9)
Respiratory events	
LO_MV_^a^	33 (24–50)
Weaning attempts	11 ± 11
Atelectasis	33 (35)
Unplanned extubations	15 (17)
Day of tracheotomy^a^	16 ± 6
Extubation failures	24 (26)
Decannulation failures	22 (23)
Successful decannulations	62 (66)
Days to successful decannulation	40 ± 19
Neuromuscular events	
Axonal polyneuropathy	14 (15)
Myopathy	3 (3)
Infusion of neuromuscular relaxants	13 (14)
Neuropsychological events	
Intracranial hypertension	26 (28)
Psychomotor agitation	15 (16)
GI and metabolic events	
Upper GI hemorrhage	16 (17)
Diarrhea	44 (47)
Ileus	31 (33)
Enteral nutrition (days)	30 ± 21
Enteral nutrition (interruptions/patient)	4 ± 3
Interruptions related to patient problems	3 ± 2
Interruptions related to tube problems	2 ± 1
Parenteral nutrition (patients)	9 (14)
Parenteral nutrition (days)	32 ± 32

Data are shown as *n *(%), or mean ± standard deviation, unless specified. ^a^Median and 0.25 to 0.75 interquartile range. APACHE, Acute Physiologic and Chronic Health Evaluation; ARDS, acute respiratory distress syndrome; CCI, chronically critically ill; ICU, intensive care unit; LO_MV_, length of mechanical ventilation; LOS_Hospital_, length of stay at the hospital; LOS_ICU_, length of stay at the ICU; MODS, multiorgan dysfunction syndrome; SOFA, sequential organ failure assessment; TISS, therapeutic intervention scoring system. GI, gastrointestinal.

**Table 4:** Sites and number of infectious episodes in CCI patients, and comparison of incidence densities with the non-CCI patients

	Patients *n *(%)	Patients with ≥2 episodes	Polimicrobial infections	Median (days) to first episode	Crude mortality	Episodes/1,000 days	Episodes/1,000 days in non-CCI
PB	59 (72)	61%	27%	9 (7–17)	39%	18^a^	7^a^
VAP	57 (70)	61%	37%	8 (6–13)	38%	17^b^	12^b^
UTI	50 (61)	72%	23%	9 (7–16)	38%	6^c^	5^c^
CRI	16 (20)	56%	8%	17 (11–39)	50%	8^d^	3^d^

^a^Number of episodes per 1,000 intensive care unit days. ^b^Number of episodes per 1,000 days of mechanical ventilation. ^c^Number of episodes per 1,000 days of urinary catheter use. ^d^Number of episodes per 1,000 days of central intravenous catheter use. CCI, chronically critically ill; CRI, catheter-related infections; PB, primary bacteremia; VAP, ventilator-associated pneumonia; UTI, urinary tract infections.
